# Identification of programmed cell death-related genes and diagnostic biomarkers in endometriosis using a machine learning and Mendelian randomization approach

**DOI:** 10.3389/fendo.2024.1372221

**Published:** 2024-08-01

**Authors:** Zi-Wei Xie, Yue He, Yu-Xin Feng, Xiao-Hong Wang

**Affiliations:** ^1^ Department of Gynecology, People's Hospital Affiliated of Fujian University of Traditional Chinese Medicine, Fuzhou, China; ^2^ First Clinical Medical College, Fujian University of Traditional Chinese Medicine, Fuzhou, China

**Keywords:** endometriosis, programmed cell death, Mendelian randomization, machine learning, gene expression, bioinformatics, single-cell analysis, molecular docking

## Abstract

**Background:**

Endometriosis (EM) is a prevalent gynecological disorder frequently associated with irregular menstruation and infertility. Programmed cell death (PCD) is pivotal in the pathophysiological mechanisms underlying EM. Despite this, the precise pathogenesis of EM remains poorly understood, leading to diagnostic delays. Consequently, identifying biomarkers associated with PCD is critical for advancing the diagnosis and treatment of EM.

**Methods:**

This study used datasets from the Gene Expression Omnibus (GEO) to identify differentially expressed genes (DEGs) following preprocessing. By cross-referencing these DEGs with genes associated with PCD, differentially expressed PCD-related genes (DPGs) were identified. Enrichment analyses for KEGG and GO pathways were conducted on these DPGs. Additionally, Mendelian randomization and machine learning techniques were applied to identify biomarkers strongly associated with EM.

**Results:**

The study identified three pivotal biomarkers: TNFSF12, AP3M1, and PDK2, and established a diagnostic model for EM based on these genes. The results revealed a marked upregulation of TNFSF12 and PDK2 in EM samples, coupled with a significant downregulation of AP3M1. Single-cell analysis further underscored the potential of TNFSF12, AP3M1, and PDK2 as biomarkers for EM. Additionally, molecular docking studies demonstrated that these genes exhibit significant binding affinities with drugs currently utilized in clinical practice.

**Conclusion:**

This study systematically elucidated the molecular characteristics of PCD in EM and identified TNFSF12, AP3M1, and PDK2 as key biomarkers. These findings provide new directions for the early diagnosis and personalized treatment of EM.

## 1 Introduction

Endometriosis (EM) is a medical disorder characterized by the presence of endometrial cells at sites beyond the uterus. It is a chronic and debilitating disorder that causes pain in the pelvic area and infertility (1, 2). According to epidemiological research, the occurrence of EM in women of reproductive age is estimated to be between 10% and 15%, impacting approximately 190 million women globally (3–5). The diagnosis of EM is frequently delayed due to uncertain etiological mechanisms, a lack of specific symptoms and of non-invasive diagnostic indicators (6). There is an average 6.7-year gap between symptom onset and diagnosis (7), with some cases taking up to 12 years to obtain an accurate diagnosis and appropriate treatment, thereby missing the optimal treatment window (8). Currently, the most reliable method for diagnosing EM is the combination of laparoscopy and histological investigation, which is widely regarded as the best approach. Nevertheless, laparoscopic surgery has inherent risks, including physical injury, adhesions, and decreased fertility (9, 10). EM has always been a hot topic in gynecological research. Moreover, exploring its pathogenesis and finding safe, effective, non-invasive diagnostic methods are crucial for patients with EM.

Programmed cell death (PCD), often referred to as regulated cell death, is a highly organized process of cellular suicide that incorporates precise signal cascades and molecular effect mechanisms. It is a conserved evolutionary process (11) that includes various forms, such as apoptosis, ferroptosis, autophagy, necroptosis, and pyroptosis (12). Under physiological conditions, PCD helps eliminate aging, damaged, or abnormal cells, thereby maintaining tissue homeostasis. However, in certain disease states, abnormal activation or inhibition of PCD can lead to pathological cell death.

Previous studies have shown that patients with EM have ectopic endometrial tissue that displays aberrant apoptotic phenomena. These phenomena may have a strong connection to the development and clinical aspects of this disease (13). Specifically, the apoptosis rate of endometrial cells in patients with EM is reduced, allowing the exfoliated cells to evade the body’s clearance mechanisms and continue growing in ectopic lesions, thereby promoting the occurrence of EM (14). Additionally, the level of autophagy in *in situ* endometrial cells of patients with EM is significantly lower than that in normal endometrial cells (15). This reduced autophagy may also decrease apoptosis, enabling cells to evade detection by the immune system and allowing endometrial cells to survive in ectopic locations. Recent studies have also found that a major defect in EM is resistance to ferroptosis, leading to abnormalities in ectopic endometrial tissue. The distribution of retrograde menstrual tissue allows the lesions to survive and settle in the abdominal cavity. Furthermore, the disruption of iron balance is believed to have a significant impact on the development of EM lesions and the resulting inflammation (16). These data indicate a strong correlation between the advancement of EM and PCD.

The relationship between PCD and EM has not been comprehensively analyzed. Therefore, this study aims to identify PCD-related biomarkers in EM using bioinformatics, Mendelian randomization, and machine learning techniques. Additionally, it seeks to establish diagnostic models and identify related molecular clusters for EM. These findings will serve as significant benchmarks for the advancement of therapeutic medications and clinical interventions.

## 2 Materials and methods

### 2.1 Data source

Gene expression data from GSE51981, GSE7305, and GSE23339 were obtained from the Gene Expression Omnibus (GEO) database (https://www.ncbi.nlm.nih.gov/geo/). The GSE51981 dataset includes 148 samples (GPL570 platform). To ensure the specificity and accuracy of the analysis, samples with other pathological conditions (non-EM) were excluded. After rigorous screening, 77 EM samples and 34 healthy control tissue samples were included as the training set for subsequent analyses. The GSE7305 dataset (GPL570 platform) includes 10 EM samples and 10 normal tissue samples, selected for validation analysis. The GSE23339 dataset (GPL6102 platform) includes 10 EM samples and 9 normal tissue samples.

FinnGen is an extensive genomics initiative that examined over 500,000 samples from Finnish biobanks to establish connections between genetic variation and health data. The objective was to comprehend the mechanisms and vulnerabilities of diseases. The project involved a partnership between Finnish academic institutes and biobanks, along with worldwide industry partners (17). We acquired publicly accessible summary statistics from the IEU Open GWAS platform, specifically the finn-b-N14_ENDOMETRIOSIS dataset. It consists of 8,288 cases of EM and 68,969 controls of European ancestry, making a total of 77,257 samples.

We also downloaded the single-cell RNA sequencing (scRNA-seq) dataset GSE213216 from the GEO database. The dataset was submitted by Kate Lawrenson on September 12, 2022, and was made public on October 11, 2022 (18). From this dataset, we obtained six normal samples and nine EM samples.

Additionally, we obtained 1,382 PCD-related genes from a previous study (19) (**Supplementary Table 1**). **Table 1** shows detailed, related information.

**Table 1:** Data source.

Name	Type	Source	Description
GSE51981	RNA-seq	GEO	77 cases of EM and 34 controls
GSE7305	RNA-seq	GEO	10 cases of EM and 10 controls
GSE23339	RNA-seq	GEO	10 cases of EM and 9 controls
GSE213216	scRNA-seq	GEO	9 cases of EM and 6 controls
PCDs	Genes	literature	Contains 1382 genes
N14_ENDOMETRIOSIS	GWAS	IEU Open GWAS	8288 cases of EM and 68969 controls

### 2.2 Identification of DEGs

The “limma” package (version 3.56.2) in R software (20) was used to identify differentially expressed genes (DEGs). DEGs were determined based on adjusted p-values below 0.05 and an absolute log2 fold change exceeding 1 between the EM and healthy control groups. A volcano plot was then generated in R using the ggplot2 package (version 3.4.4) to graphically represent DEGs. This plot demonstrates both the extent of gene expression variations and their statistical significance. The R package ggvenn (version 0.1.10) (21) was utilized to conduct an intersection analysis between DEGs and programmed cell death related genes (PCDs) to find differentially expressed PCDs (DPGs).

### 2.3 GO and KEGG analysis

To ascertain the functions of DPGs in EM, we performed GO and KEGG pathway enrichment analyses using the clusterProfiler package (version 4.8.3) in R (22). The significance level was established at a p-value of less than 0.05. GO analysis encompasses three essential categories: cellular components (CC), molecular functions (MF), and biological processes (BP), which are vital for scrutinizing physiological operations (21). The researchers utilized KEGG analysis to investigate possible pathways (22). The R tools, ggplot and GOplot, were utilized to visually represent the outcomes of the GO and KEGG studies.

### 2.4 Identifying candidate genes

To investigate the cause-and-effect relationship between DPGs and EM, DPGs were considered the independent variables and EM the dependent variable in the Mendelian randomization (MR) analysis. The R package ‘TwoSampleMR’ (version 0.5.6) (23) was used to identify single nucleotide polymorphisms (SNPs) that are strongly linked to the exposure factors and can be used as instrumental variables (IVs) (P < 5×10^^−8^). IVs exhibiting significant linkage disequilibrium (LD) (r² < 0.001, kb = 10000) were subsequently eliminated. The ‘TwoSampleMR’ package’s mv_harmonise_data function was employed to standardize effect alleles and effect sizes, whereas the mv_lasso_feature_selection function was utilized to remove collinearity and choose variables. Several MR techniques were employed to validate the causal association between important target genes and EM, such as Wald ratio, inverse variance weighting (IVW) (24), MR Egger (25). When there was only one eQTL available for a gene, the Wald ratio approach was utilized. For genes with two or more genetic tools, the IVW method was given priority (26). Special emphasis was placed on genes with p-values below 0.05. Furthermore, using Cochran’s Q test, heterogeneity tests were performed to assess the presence of heterogeneity, with P-values less than 0.05 indicating the presence of heterogeneity. Additionally, Egger’s regression intercept was used for horizontal pleiotropy tests, with P-values less than 0.05 indicating evidence of horizontal pleiotropy. The expression patterns of these genes in the GSE51981 training set were evaluated to validate the final candidate genes.

### 2.5 Identification of biomarkers and construction of diagnostic models

Random forest (RF) (27), support vector machine (SVM) (28), LightGBM (29), gradient boosting decision tree (GBDT) (30), and XGBoost (31) were used to accurately identify biomarkers associated with EM by taking the intersection of the results from these different machine learning methods. Subsequently, multivariable logistic regression analysis was conducted using the rms package (version 6.7.1) in R (32), and a nomogram model for diagnosing EM was constructed based on the biomarker data. The “total score” of the model was calculated by aggregating the scores of each predictor, with each predictor being allocated a distinct value. The diagnostic accuracy of the nomogram model was assessed by analyzing ROC curves and AUC values obtained from both the training and validation sets. This analysis was conducted to confirm the model’s diagnostic effectiveness. The evaluation of the ROC curve was conducted using the pROC package (version 1.18.5) in the R programming language (33). Additionally, the accuracy and practical value of the diagnostic model were evaluated by performing decision curve analysis and calibration curve analysis using the rms package (version 6.7.1) (32). Furthermore, the ggpubr package (version 0.6.0) in R was used to provide the expression levels of biomarkers in both the training and validation sets.

### 2.6 Single cell RNA sequencing

The scRNA-seq data was analyzed using the Seurat tool (version 5.0.1) in R (34). Cells that exhibited less than 200 gene expressions or genes that were expressed in less than three cells were eliminated. Furthermore, cells with Unique Molecular Identifier (UMI) counts below 300 or above 2500, cells that expressed mitochondrial genes in over 10% of the cell population, and cells that expressed hemoglobin genes in over 3% of the cell population were excluded. The Seurat package’s “NormalizeData” function was employed for data normalization, which was subsequently followed by batch correction and dimensionality reduction using the Harmony package (version 0.1.1) in R (35). At a resolution of 0.5, 20 principal components (PCs) were used to identify cell clusters. The “FindAllMarkers” function in Seurat was utilized to identify differentially expressed genes for each cluster, using the following parameters: “min.pct = 0.25” and “logfc.threshold = 0.25”. Afterwards, the SingleR package (version 2.4.0) (36) was utilized along with known cell type marker genes to label each cell cluster. Ultimately, the analysis focused on the distribution and expression patterns of these genes across various cell types.

### 2.7 Consensus cluster analysis

The training set of 77 EM samples underwent unsupervised hierarchical clustering analysis using the ConsensusClusterPlus package (version 1.64.0) in R (37). The analysis involved 1000 iterations with a sample rate of 80%. The following criteria were used as the basis for clustering: 1) the cumulative distribution function (CDF) curve flattens and grows gradually; 2) no cluster contains a small number of samples; 3) the Δ area decreases to its maximum; and 4) after clustering, inter-cluster correlation decreases, while intra-cluster correlation increases.

### 2.8 Evaluating the immune infiltration of the sub-clusters

To evaluate the presence of immune cells in the sub-clusters, we utilized the GSVA package (version) (38) and the GSEAbase package (version) (39) in R to measure the proportional abundance of immune cells within these sub-clusters. Enrichment scores were compared among different subgroups and visualized using the ggplot2 package (version 3.4.4).

### 2.9 Protein-ligand interaction analysis

We obtained the 3D structures of three key target proteins from UniProt (https://www.uniprot.org/). The chemical structures of Dienogest, Goserelin, and Danazol in Structure Data File (SDF) format were sourced from PubChem (https://pubchem.ncbi.nlm.nih.gov/). These structures were converted to mol2 format using the OpenBabel program (40). The protein crystal structures were acquired from the Protein Data Bank (PDB) and underwent dehydration and hydrogenation using PYMOL (41). The converted protein receptors and small-molecule ligands were used in molecular docking experiments with AutoDock software. A visualization of the docking results was created using PyMOL.

### 2.10 Statistical analyses

The bioinformatics analyses and execution of all R programs were performed using R software (version 4.3.1). The means of normally distributed variables between two groups were compared using unpaired Student’s t-tests. If the data did not follow a normal distribution, the Wilcoxon test was used to compare them. Significance levels were denoted as ∗P < 0.05, ∗∗P < 0.01, and ∗∗∗P < 0.001.

## 3 Results

### 3.1 Identification of DPGs between EM and normal controls

**Figure 1:**
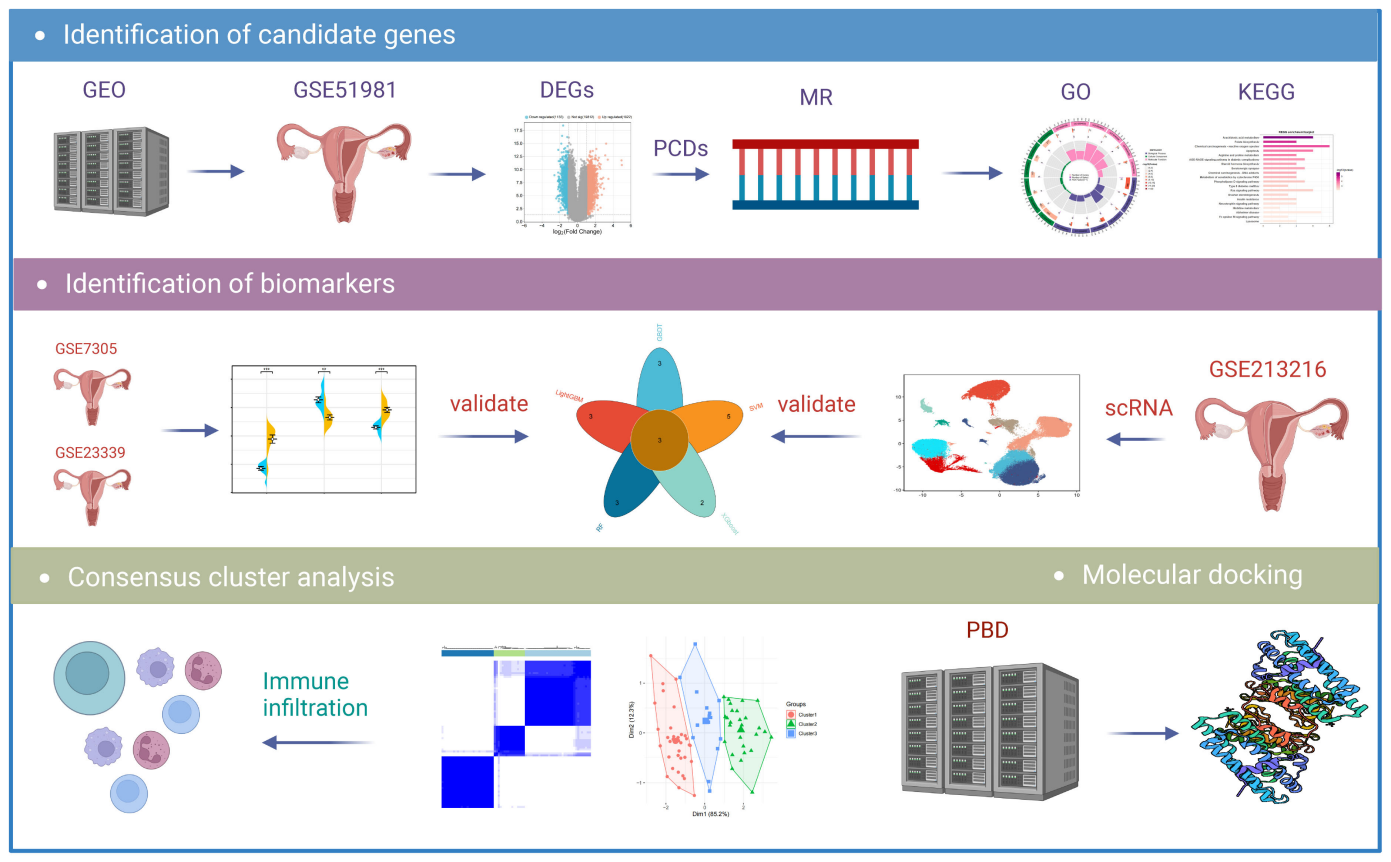
Flowchart of the research. DEGs, differentially expressed genes; MR, Mendelian randomization; GO, Gene Ontology; KEGG, Kyoto Encyclopedia of Genes and Genomes; scRNA, Single-Cell RNA Sequencing Analysis.

**Figure 2:**
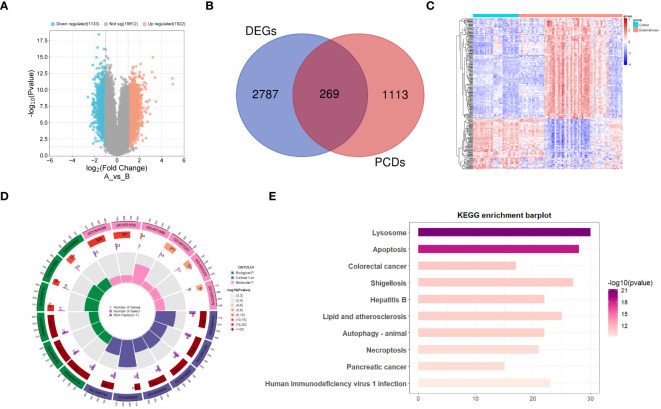
Differential expression analysis of programmed cell death genes in endometriosis **(A)** Volcano plot of differentially expressed genes in endometriosis. **(B)** Venn diagram of differentially expressed genes and genes related to programmed cell death, showing their intersecting genes. **(C)** Heatmap of DPGs. **(D)** Gene Ontology enrichment analysis of DPGs. **(E)** KEGG pathway enrichment analysis of DPGs.

**Figure 1** illustrates the detailed workflow of this study. A total of 3055 differentially expressed genes (DEGs) related to EM were identified, including 1922 upregulated genes and 1133 downregulated genes (**Supplementary Table S2**). These DEGs were visualized using a volcano plot (**Figure 2A**). Subsequently, by intersecting the 1382 known PCD-related genes with the DEGs, we identified 269 DPGs (**Figure 2B**). The expression differences of these DPGs between the EM group and healthy controls were visually represented using a heatmap (**Figure 2C**).

### 3.2 Functional annotation and pathway enrichment of DPGs

Functional annotation and pathway enrichment analyses were performed on the DPGs. The results of the GO enrichment analysis indicate that these genes are involved in multiple signaling pathways associated with PCD (**Figure 2D**). The pathways encompassed are the intrinsic apoptotic signaling pathway, regulation of apoptotic signaling pathway, regulation of autophagy, macroautophagy, intrinsic apoptotic signaling pathway triggered by DNA damage, extrinsic apoptotic signaling pathway, mitochondrial structure organization, apoptotic mitochondrial changes, negative regulation of apoptotic signaling pathway, and regulation of intrinsic apoptotic signaling pathway. The KEGG pathway enrichment analysis (**Figure 2E**) indicated that these genes are involved in pathways, such as lysosome, apoptosis, autophagy, and necroptosis.

### 3.3 Identification of candidate genes

**Figure 3:**
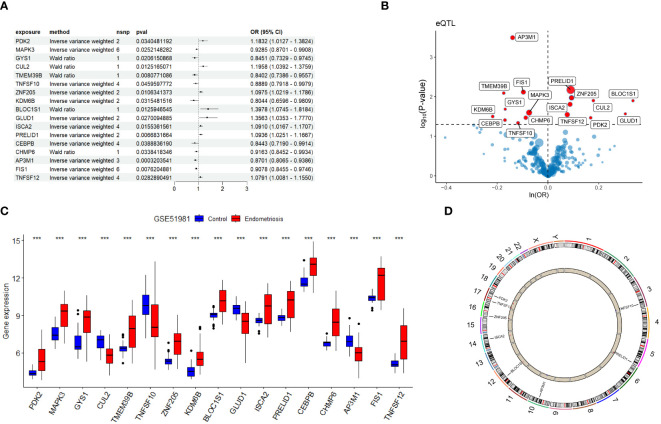
Identification of candidate genes. **(A)** Forest plot presenting Mendelian randomization analysis results for 17 genes. **(B)** Volcano plot illustrating Mendelian randomization analysis of DPGs. **(C)** Gene expression levels of 17 candidate genes in the GSE51981 dataset. **(D)** Chromosomal distribution of copy number variation locations for eight candidate genes. Significance levels were denoted as ^∗^P < 0.05, ^∗∗^P < 0.01, and ^∗∗∗^P < 0.001.

In this study, MR analysis was performed on 269 genes. Using Wald ratio and IVW methods, we identified 17 genes with statistical significance (P-values < 0.05) under strict selection criteria. During this process, we paid particular attention to eliminating interference that may arise from pleiotropy and heterogeneity, ensuring the accuracy and reliability of the analysis results. Among these 17 genes, eight had odds ratios (OR) greater than 1, while the other nine had OR values less than 1 (**Figures 3A, B**). We then observed the expression patterns of these genes in the GSE51981 training set. The results showed that in the EM group, 13 genes had significantly increased expression levels, while four genes had significantly decreased expression levels (**Figure 3C**). Based on these findings, we inferred that genes with OR greater than 1 and increased expression levels in the EM group are positively correlated with EM, whereas those with OR less than 1 and decreased expression levels in the EM group may be negatively correlated. Further analysis identified six potential harmful genes and two potential protective genes, which were determined to be candidate genes. The copy number variation (CNV) positions of these eight candidate genes were mapped to their respective chromosomal locations (**Figure 3D**).

### 3.4 Identification and validation of biomarkers for EM

**Figure 4:**
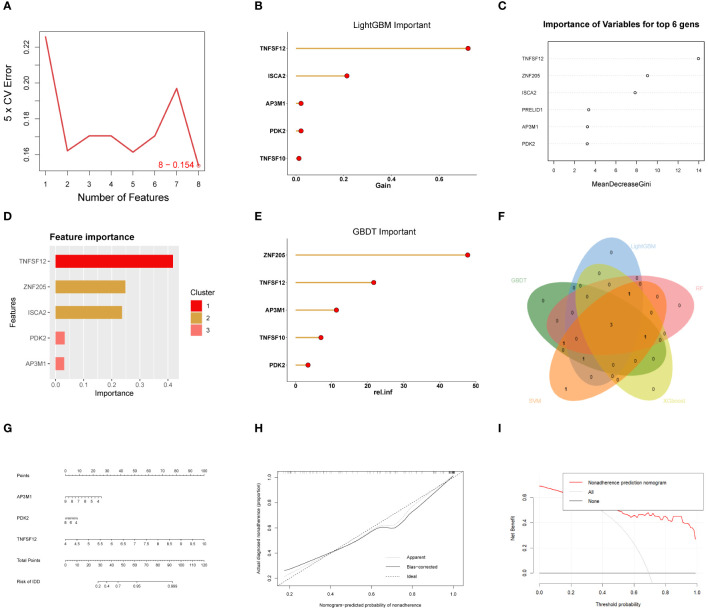
Machine learning-based biomarker identification. **(A)** SVM identified eight biomarkers. **(B)** LightGBM identified six biomarkers. **(C)** RF identified six biomarkers. **(D)** XGBoost identified five biomarkers. **(E)** GBDT identified six biomarkers. **(F)** Venn diagram of biomarkers identified using different machine learning methods, showing their intersecting biomarkers. **(G-I)** Construction of EM Diagnostic Model.

In this study, various machine learning techniques were used for gene selection. SVM identified eight genes (**Figure 4A**), While LightGBM identified five genes, RF identified six genes. (**Figures 4B, C**). XGBoost identified five genes (**Figure 4D**), and GBDT identified five genes (**Figure 4E**). Through intersection analysis of these results, three key genes were ultimately identified: TNFSF12, AP3M1, and PDK2 (**Figure 4F**). A model was constructed based on these three key diagnostic genes to predict the risk of EM, and its overall performance was evaluated (**Figure 4G**). The predictive accuracy of the model was validated using calibration curves (**Figure 4H**) and decision curve analysis (DCA) (**Figure 4I**). ROC analysis showed that the combined diagnostic AUC value of these three genes was 0.9, indicating its high accuracy in diagnosing EM. Additionally, the model was evaluated using validation sets GSE7305 and GSE23339, with ROC analysis showing AUC values of 0.94 and 0.88, respectively, highlighting the model’s stability and reliability across different datasets.

**Figure 5:**
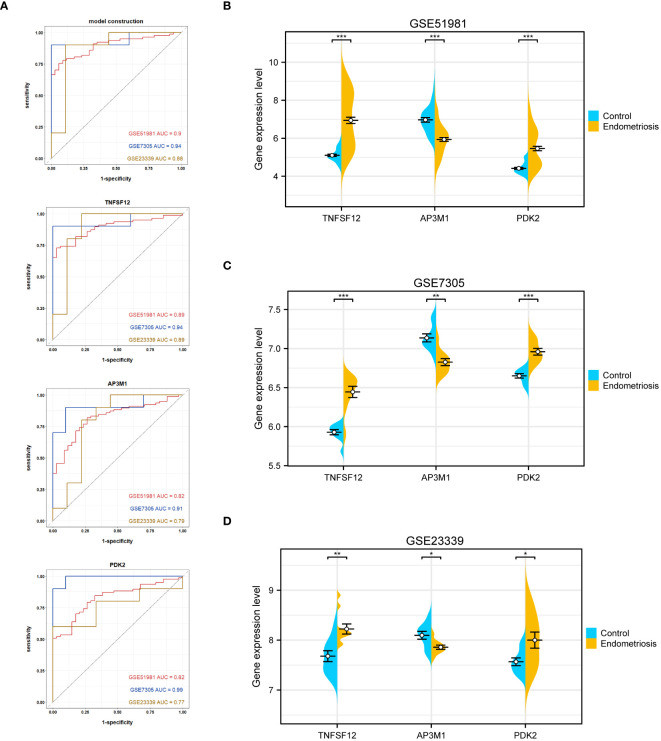
Validation of the diagnostic value of the diagnostic model and biomarkers. **(A)** ROC evaluation of the diagnostic value of the model and biomarkers. **(B)** Gene expression levels of biomarkers in GSE51981. **(C)** Gene expression levels of biomarkers in GSE7305. **(D)** Gene expression levels of biomarkers in GSE23339. Significance levels were denoted as ^∗^P < 0.05, ^∗∗^P < 0.01, and ^∗∗∗^P < 0.001.

Regarding individual gene performance, TNFSF12 demonstrated an AUC of 0.89 in the training dataset GSE51981 and achieved AUC values of 0.94 and 0.89 in the validation datasets GSE7305 and GSE23339, respectively. Regarding AP3M1, the AUC was 0.82 in the training set, with corresponding values of 0.91 and 0.79 in the validation sets GSE7305 and GSE23339, respectively. PDK2 showed an AUC of 0.82 in the training dataset and attained values of 0.99 and 0.77 in the validation datasets GSE7305 and GSE23339 (**Figure 5A**). These data emphasize the potential of TNFSF12, AP3M1, and PDK2 as diagnostic biomarkers for EM. Additionally, we analyzed the expression differences of these three genes in both the training and validation sets. In the training set GSE51981, TNFSF12 and PDK2 showed significantly increased expression in EM samples compared to healthy endometrial samples, whereas AP3M1 was significantly decreased in EM samples (**Figures 5B**). These expression patterns were validated in the validation sets GSE7305 and GSE23339 (**Figures 5C, D**),consistent with the findings in the training set. These results further support the potential of TNFSF12, AP3M1, and PDK2 as diagnostic biomarkers for EM.

### 3.5 Biomarkers analysis in scRNA-seq data

**Figure 6:**
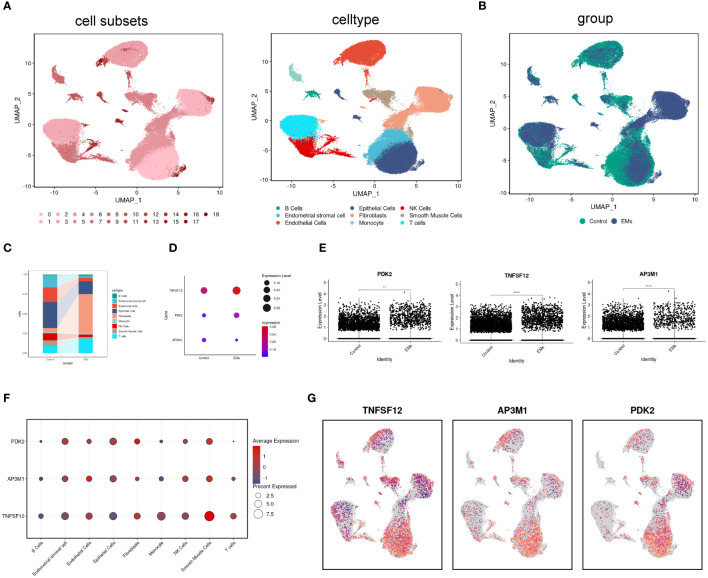
Distribution and expression of biomarkers in single cells. **(A)** UMAP plot showing 17 identified cell subpopulations and nine cell types. **(B)** UMAP plot displaying cell distribution in normal and EM samples. **(C)** Changes in the proportions of nine cell subpopulations in normal and EM samples. **(D, E)** Expression levels of biomarkers in normal and EM samples. **(F, G)** Distribution of biomarkers across nine cell subpopulations.

scRNA-seq analysis was performed on tissue samples from patients with EM and from participants in the control group, initially comprising 144,476 cells (42,641 from EM samples and 101,835 from control samples). After stringent data quality control, 90,615 cells were retained for subsequent analysis (32,024 cells from EM samples and 58,591 cells from control samples). High-dimensional data dimensionality reduction and clustering analysis were performed using Uniform Manifold Approximation and Projection (UMAP), successfully identifying 19 different cell subpopulations. The cell types were identified as T cells, endometrial stromal cells, NK cells, monocytes, endothelial cells, smooth muscle cells, fibroblasts, B cells, epithelial cells (**Figure 6A**). Comparative analysis of cell composition differences between EM samples and control groups indicated an increase in B cells, fibroblasts, and T cells in EM samples, while the proportions of endometrial stromal cells, endothelial cells, epithelial cells, NK cells, and smooth muscle cells decreased (**Figures 6B, C**). In this study, we particularly focused on the distribution and expression patterns of the core genes TNFSF12, AP3M1, and PDK2.

The analysis results showed that in the EM group, the expression levels of TNFSF12 and PDK2 were significantly elevated, while AP3M1 exhibited a significant decreasing trend. These differences were highly significant statistically and consistent with previous RNA sequencing analysis results (**Figures 6D, E**). Subsequently, we conducted a detailed analysis of cell type distribution to gain deeper insights into the expression patterns of these genes. We found that TNFSF12 was primarily distributed in smooth muscle cells and fibroblasts, with high expression levels. Similarly, AP3M1 was mainly expressed in endothelial cells and smooth muscle cells, whereas PDK2’s high expression was limited to fibroblasts (**Figures 6F, G**). These findings are significant, highlighting the potential of TNFSF12, AP3M1, and PDK2 as biomarkers.

### 3.6 Identification of biomarker sub-clusters in EM

**Figure 7:**
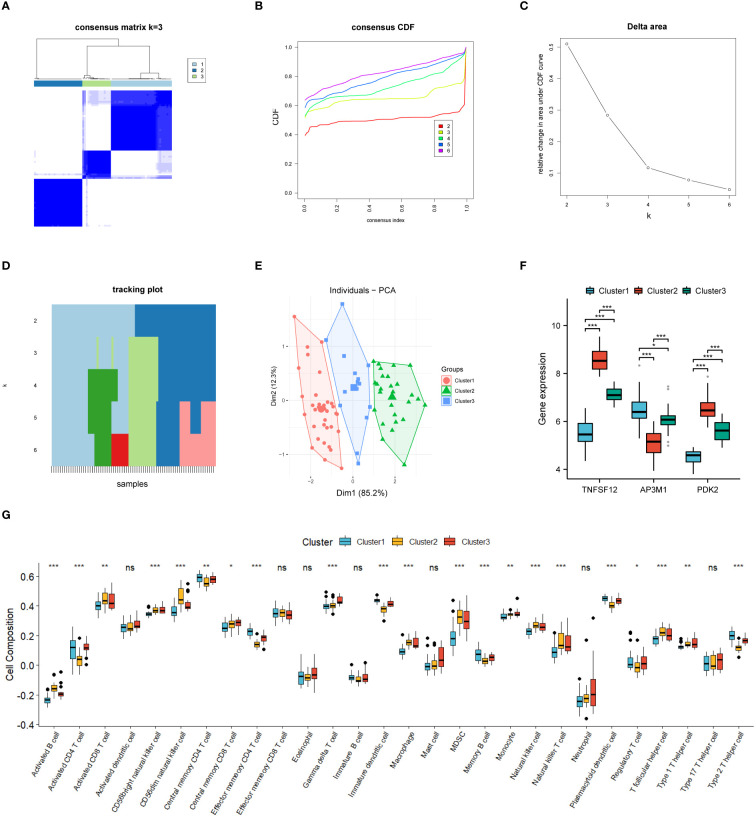
Consensus cluster analysis. **(A–E)** Consensus clustering analysis based on biomarkers identified three subclusters. **(F)** Gene expression levels of three biomarkers across the three subclusters. **(G)** Immune cell infiltration abundance in the three subclusters. Significance levels were denoted as follows: *P < 0.05, **P < 0.01, and ***P < 0.001. NS indicates non-significant results with P > 0.05.

In this study, we divided 77 EM samples into different groups based on the expression profiles of TNFSF12, AP3M1, and PDK2. Using an unsupervised hierarchical clustering algorithm, the most stable clustering result was obtained when the k-value was set to 3 (k=3) (**Figures 7A–D**). Subsequently, principal component analysis (PCA) revealed significant specificity in gene expression patterns among different sub-clusters, as validated by the PCA plot (**Figure 7E**). Additionally, we created a series of box plots (**Figure 7F**), illustrating the expression levels of these genes in different sub-clusters and their significant differences.

### 3.7 Discrimination of immune infiltration characteristics among the sub-clusters

Based on our subgroup analysis of EM samples, single-sample gene set enrichment analysis (ssGSEA) was used to assess immune infiltration in different subgroups, particularly focusing on significant differences with P-values less than 0.05. The immune infiltration analysis results (**Figure 7G**) showed significant changes in the immune microenvironment among the different subgroups. Specifically, in Cluster 1, we observed higher abundances of certain immune cell types, such as central memory CD4+ T cells, effector memory CD4+ T cells, memory B cells, plasmacytoid dendritic cells, and type 2 helper T cells. In Cluster 2, the numbers of activated B cells, CD56dim natural killer cells, and helper T cells significantly increased. Meanwhile, in Cluster 3, the infiltration of γ-δ T cells was relatively higher.

### 3.8 Molecular docking of biomarkers

**Figure 8:**
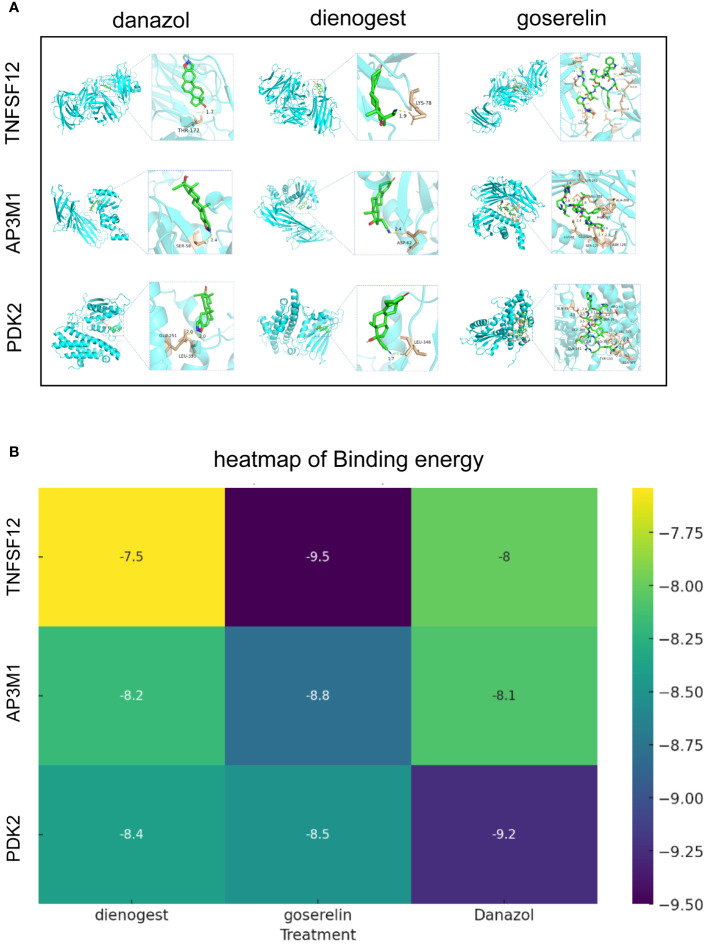
Molecular docking results of biomarkers and clinical drugs. **(A)** Molecular Docking Visualization of Biomarkers and Clinical Drugs. **(B)** Heatmap of Binding Energies from Molecular Docking Visualization of Biomarkers and Clinical Drugs.

Globally, dienogest, danazol, and goserelin are widely used for treating EM. To further investigate the interactions between these drugs and biomarkers, we conducted detailed studies using advanced molecular docking techniques. In this process, we focused on evaluating the binding capabilities of these drug molecules with the three biomarkers, quantifying this capability by precisely calculating binding energies. Typically, lower binding energy values indicate more stable binding conformations. Our molecular docking analysis demonstrated significant binding affinities between the three biomarkers and dienogest, danazol, and goserelin. All docking binding energies were below −7.54 kcal/mol, indicating that these drugs can effectively bind to biomarkers naturally. These results not only confirm the molecular specificity of these drugs but also provide important insights into their mechanisms of action in treating EM (**Figures 8A, B**).

## 4 Discussion

EM is a multifaceted gynecological disease that affects the entire body (4, 5). Because of its rising occurrence, frequent relapse, and notable shortcomings in clinical diagnosis and treatment, it has become a subject of academic research in recent years (6, 9). Nevertheless, the absence of precise biomarkers that can be used to detect EM has significantly impeded its prompt diagnosis, thereby necessitating research for the identification of reliable molecular biomarkers for a correct diagnosis. This study aimed to examine the function and diagnostic importance of PCDs in EM.

Through the intersection of DEGs and PCDs, we identified a total of 269 DPGs. Enrichment analysis of these genes revealed that multiple key biological pathways play important roles in EM. Apoptosis, a form of PCD, plays a significant role in EM. Compared to cells from women without EM, ectopic endometrial stromal cells (EESC) and eutopic endometrial stromal cells (EuESC) from patients with EM exhibited enhanced survival capabilities. Several studies have demonstrated that endometrial cells from patients with EM are less sensitive to apoptosis than those from healthy controls (14, 42). It has been shown that EESCs exhibit higher expression levels of the anti-apoptotic genes Bcl-2 and Bcl-xL compared to normal endometrial cells (CESCs) and diseased endometrial cells (EuESCs). The upregulation of these genes inhibits apoptosis, thereby increasing the survival capacity of ectopic endometrial cells. Moreover, the expression of the apoptosis-related gene Caspase-3 is significantly reduced in EESCs, further supporting their resistance to apoptosis (43). The p53 and TNF pathways play crucial roles in cell cycle regulation and apoptosis. The inhibition of p53 signaling pathway activity and the dysregulation of TNF signaling pathway activity also lead to reduced apoptosis, thereby promoting the progression of EM (44, 45).

Necroptosis, a form of programmed necrosis, that distinct from apoptosis, is an active cell death process executed through specific molecular mechanisms. Necroptosis signaling is closely related to various signaling pathways involved in physiological responses, such as inflammation, immune response, and tissue homeostasis maintenance (46). Studies have shown that the expression of necroptosis-related genes is associated with the pathological processes of EM, particularly in terms of inflammatory response and immune cell infiltration (15, 47). These findings suggest that PCDs play important roles in EM, affecting cell survival, proliferation, and apoptosis through various mechanisms, thereby contributing to the progression of EM.

Due to the swift advancement of artificial intelligence (AI), machine learning algorithms can be used to effectively differentiate and analyze complex feature data. Consequently, they are extensively employed in the discovery and screening of pivotal genes. MR is a strategy used to infer causality based on genetic variation. It has gained significant popularity in medical and scientific research in recent years. By using genetic variants as instrumental variables, MR avoids common confounding factors and reverse causation issues, enabling a more accurate evaluation of the causal effects of genes on diseases (48). Initially, we used MR to validate whether the DPGs were indeed causally related, identifying eight key candidate genes from the 269 DPGs. Subsequently, we integrated five different machine learning models to analyze the expression profiles of the candidate genes, precisely identifying three biomarkers (TNFSF12, AP3M1, and PDK2).

The results of the Mendelian randomization analysis indicated that TNFSF12 and PDK2 were positively correlated with EM in genetic predictions, whereas AP3M1 showed an inverse relationship. ROC curve assessments demonstrated that these three genes had good diagnostic performance both in combination and individually in diagnosing EM, highlighting their potential as biomarkers. Further validation of these results was achieved through transcriptome mRNA expression level analysis, using data from both the training and validation sets, showing significantly elevated levels of TNFSF12 and PDK2 in the EM group, while AP3M1 was significantly reduced. Through various analyses, we confirmed the critical roles of these biomarkers in EM, further establishing their potential as diagnostic tools and therapeutic targets.

TNFSF12, also known as TWEAK or CD255, is a member of the tumor necrosis factor (TNF) superfamily and is an important pro-inflammatory cytokine widely expressed in various tissues and cells, participating in multiple physiological and pathological processes (49). Although there are no direct reports linking TNFSF12 to EM, existing studies suggest that the interaction between TNFSF12 and its receptor Fn14 can activate several signaling pathways, including the classical and alternative NF-κB pathways, leading to the production and release of inflammatory cytokines (50). This can result in exacerbated local inflammatory responses. These inflammatory factors might accumulate in endometriotic lesions, intensifying local inflammation and promoting the maintenance and expansion of the lesions. Additionally, the TWEAK-Fn14 interaction can regulate cell survival and proliferation through multiple signaling pathways, including PI3K/Akt and MAPK (51, 52) The activation of these pathways can upregulate the expression of anti-apoptotic genes, contributing to the occurrence and progression of EM. TNFSF12 also modulates the functions of immune cells, such as NK cells and T cells. TWEAK can act on embryonic stem cells to inhibit Th1 immune activation by suppressing the activity of NK cells (49). Studies have shown that in patients with EM, the function of NK cells is impaired, with significantly reduced cytotoxic capabilities. This dysfunction might enable endometriotic tissue to evade immune system clearance, facilitating the invasion and sustained growth of ectopic endometrial cells (53). Furthermore, TNFSF12 can promote the expression of angiogenic factors (51), potentially aiding in the formation of new blood vessels in the lesion area, thereby providing sufficient nutrients and oxygen to ectopic endometrial cells, which further promotes lesion expansion.

AP3M1 is a key subunit of the AP-3 complex, which is primarily responsible for intracellular vesicle transport from the Golgi apparatus to lysosomes and related organelles (54). This regulation is crucial for maintaining cell function and structural integrity. Studies have shown a negative correlation between AP3M1 and EM risk through genome-wide MR and colocalization analysis (55), which is consistent with our findings. Previous studies have confirmed that the loss of AP-3 complex function negatively impacts the normal cytotoxic activity of NK and CTL cells. The absence of the AP-3 complex leads to degranulation defects in these cells, thereby weakening their cytotoxic function (56). Since EM is associated with weakened NK cell cytotoxicity, we speculate that the loss of AP3M1 may lead to dysfunction of the AP-3 complex, affecting NK cell toxicity and promoting the progression of EM.

PDK2 is a member of the pyruvate dehydrogenase kinase family and is expressed in various tissues throughout the body (57). PDKs, including PDK2, are key metabolic regulatory enzymes that inhibit the activity of pyruvate dehydrogenase (PDH) through phosphorylation, altering the metabolic state of cells. This mechanism is expressed in various tissues and promotes aerobic glycolysis in tumor cells and other pathological states, supporting cell survival in hypoxic environments (58). PDK2 plays a crucial role in balancing glycolysis and oxidative phosphorylation by regulating the activity of the pyruvate dehydrogenase complex (PDHC). When PDK2 expression is elevated, cell metabolism undergoes reprogramming similar to that of tumor cells (59). Specifically, PDK2 inhibits PDH activity, thereby preventing pyruvate from adequately entering the TCA cycle, leading to reduced mitochondrial membrane potential and decreased reactive oxygen species (ROS) production. This environment reduces the occurrence of apoptosis, potentially helping ectopic endometrial cells evade PCD.

In single-cell analysis, we observed significantly elevated expression levels of TNFSF12 and PDK2 in EM, whereas AP3M1 expression was significantly reduced. This finding further supports our previous conclusions. The application of molecular docking technology is crucial for understanding the interactions between clinical drugs and biomarkers, using limited resources to identify potential strong drugs. Dienogest, goserelin, and danazol are commonly used non-surgical treatments for EM (60, 61). Through molecular docking, we assessed the binding abilities of dienogest, goserelin, and danazol to these biomarkers. The calculated binding energies indicated significant affinity between these clinical drugs and the biomarkers, suggesting that TNFSF12, AP3M1, and PDK2 could serve as potential targets for dienogest, goserelin, and danazol. The specific regulatory mechanisms of dienogest, goserelin, and danazol on TNFSF12, AP3M1, and PDK2 still require further investigation.

Additionally, unsupervised clustering analysis indicated that, based on the expression levels of biomarkers, different patients with EM could be divided into three distinct subgroups. For the different subgroups, we also conducted immune cell abundance and infiltration analyses. The differences in immune infiltration characteristics among the various subgroups of EM may reflect the diverse roles and mechanisms of immune regulation within these subgroups. It is well known that EM exhibits heterogeneity in clinical presentation and treatment, reflecting its complex etiology, suggesting that stratified management plans based on its characteristics may yield better outcomes.

Although this study employed multi-omics methods and validated the roles of these biomarkers in EM at multiple levels, some limitations still need to be addressed. First, this study is entirely based on bioinformatics analysis, emphasizing the necessity for subsequent experimental validation. Furthermore, the conclusions were derived from a restricted number of EM cases, highlighting the necessity for more extensive patient research to bolster the dependability of the findings. The diagnostic model established in this study, known as the EM model, also needs to undergo additional scrutiny and external validation before it can be potentially applied in clinical settings.

## 5 Conclusions

In summary, this study systematically revealed the molecular characteristics of PCD in EM and identified three key biomarkers: TNFSF12, AP3M1, and PDK2. Through MR analysis, we confirmed the genetic causal relationships between these genes and EM. Multi-dataset validation and single-cell analysis further substantiated the significant expression differences of these genes in ectopic endometrial tissue. Additionally, molecular docking analysis demonstrated significant binding affinities between these genes and clinically used drugs, highlighting their potential in diagnosis and treatment. These findings suggest that TNFSF12, AP3M1, and PDK2 could be potential diagnostic biomarkers and therapeutic targets for EM.

## Data availability statement

The datasets presented in this study can be found in online repositories. GSE51981, GSE7305, GSE23339, and GSE213216 are available at https://www.ncbi.nlm.nih.gov/. The finn-b-N14_ENDOMETRIOSIS dataset is available at https://gwas.mrcieu.ac.uk/.

## Ethics statement

Ethical approval was not required for the study involving humans in accordance with the local legislation and institutional requirements. Written informed consent to participate in this study was not required from the participants or the participants’ legal guardians/next of kin in accordance with the national legislation and the institutional requirements.

## Author contributions

Z-WX: Conceptualization, Data curation, Formal analysis, Funding acquisition, Investigation, Methodology, Project administration, Resources, Software, Supervision, Validation, Visualization, Writing – original draft, Writing – review & editing. YH: Conceptualization, Data curation, Formal analysis, Investigation, Methodology, Project administration, Software, Supervision, Validation, Writing – original draft, Writing – review & editing. Y-XF: Data curation, Formal analysis, Investigation, Methodology, Project administration, Resources, Software, Supervision, Validation, Visualization, Writing – original draft, Writing – review & editing. X-HW: Data curation, Funding acquisition, Investigation, Software, Supervision, Writing – review & editing.
