# Supplementary figures and images for: Identification of programmed cell death-related genes and diagnostic biomarkers in endometriosis using a machine learning and Mendelian randomization approach
Source: Front Endocrinol (Lausanne). 2024 Aug 1;15:1372221. doi: 10.3389/fendo.2024.1372221 (PMC11324423; doi:10.3389/fendo.2024.1372221)

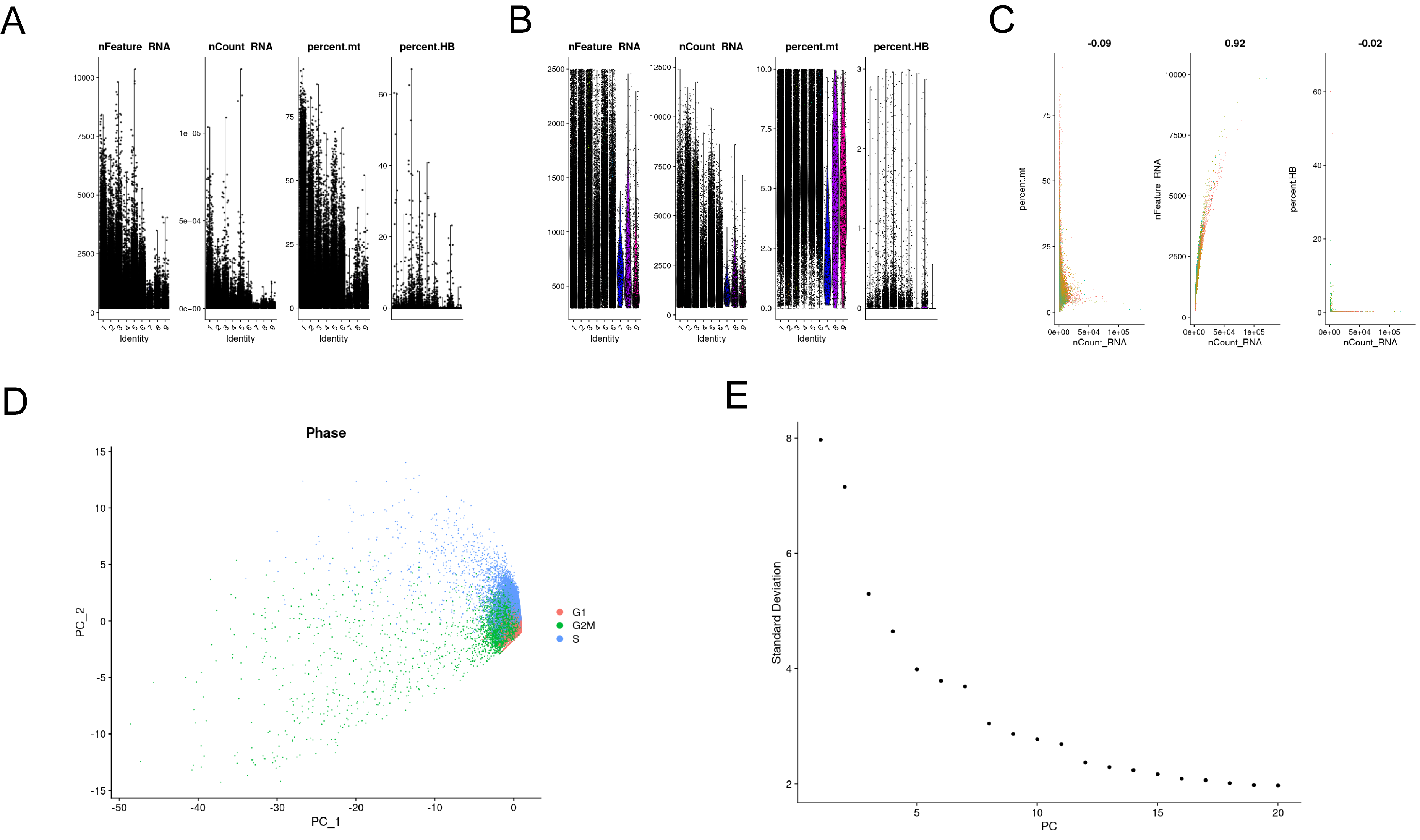

Supplement: Supplementary file 1 [file Image_1.tif]
